# Evaluation of Acid-Modified Ethiopian Potato (*Plectranthus edulis*) Starch as Directly Compressible Tablet Excipient

**DOI:** 10.1155/2020/9325173

**Published:** 2020-04-06

**Authors:** Amsalu Gulla, Afewerk Getachew, Tsadkan Gebremeskel Haile, Fantahun Molla

**Affiliations:** ^1^Department of Pharmacy, School of Health and Medical Sciences, Dilla University, Dilla, Ethiopia; ^2^Department of Pharmaceutics, School of Pharmacy, College of Health Sciences, Mekelle University, Mekelle, Ethiopia

## Abstract

Ethiopian potato is one of the tuber-bearing members of the family Lamiaceae. It is an indigenous crop in Ethiopia and important source of starch. Unprocessed native starches are structurally weak and functionally restricted for application in pharmaceutical technologies. Consequently, starch is usually modified either chemically or physically to make it convenient for industrial use. The aim of the study was to prepare and characterize acid-modified Ethiopian potato starch (AMEPS) and evaluate its functionality as a direct compressible excipient in tablet formulations. The extracted starch from Ethiopian potato tuber was modified using 6% HCl concentration for 8 days, then dried using oven and spray drying techniques, and subsequently evaluated and compared with the native Ethiopian potato starch (NEPS) and S1500® as a direct compressible excipient. Acid modification of the NEPS decreased the moisture content and swelling power while increased the percent solubility. The X-ray diffraction revealed that both the NEPS and AMEPS have B-type crystal patterns. The AMEPS showed improved flowability compared to the NEPS. This improvement was further enhanced by the spray drying process. The compactability study revealed that the tensile strength of spray-dried AMEPS (16.76 kg/cm^2^) was significantly higher than that of the spray-dried NEPS (7.07 kg/cm^2^) and S1500® (11.66 kg/cm^2^). The AMEPS was less sensitive to lubricants compared to the NEPS and Starch 1500®. Similarly, the dilution potential of the AMEPS was superior to the NEPS and S1500®. The AMEPS accommodated up to 50% of paracetamol while the NEPS and S1500® were able to hold only up to 30%. Pharmacopoeial specifications for disintegration and dissolution were met by the paracetamol tablets prepared by AMEPS. Thus, considering all the results obtained, spray-dried AMEPS could be a potential alternative directly compressible tablet excipient.

## 1. Introduction

Today's technologic desire is to simplify the manufacturing processes while at the same time getting cheap and quality products. Regarding tablet manufacturing, direct compression (DC) had become one approach that simplifies the tablet manufacturing process [1]. However, the availability of a small number of excipients for DC of tablets has been the challenge [2]. Thus, there is a growing need in developing new multifunctional DC excipients [3]. A potential source of these multifunctional DC excipients is naturally existing polymeric materials such as starch.

**Figure 1:**
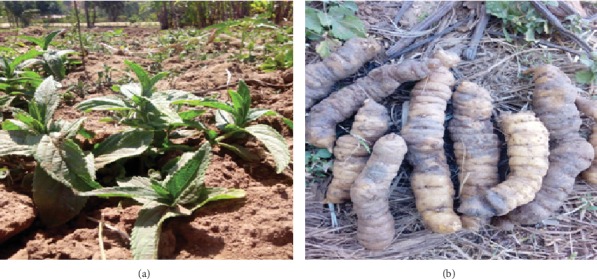
Ethiopian potato plant (a) and its tuber (b) (photograph taken by Amsalu G., 2019).

Starch can generally be recognized as the most common pharmaceutical excipient [4, 5] which has versatile application in various dosage forms. In this regard, there is a continuous need for the development of new starch excipients with suitable properties to meet the special needs of drug formulators [6]. Many studies have shown that Ethiopia has numerous plant species that can be used as an alternative source of starch for various pharmaceutical applications (Gebre-Mariam and Schmidt, 1996, [7–10]). Hence, it is useful to produce starch-based excipients from cheap indigenous sources such as the Ethiopian potato tuber (Figure 1).

Ethiopian potato tubers have a starch yield value of about 80.4% on a dry weight basis [8]. However, the native Ethiopian potato starch (NEPS) is not suitable for many pharmaceutical applications including DC. This is due to the inherent weaknesses of flow, compressibility, and lubricant sensitivity [11]. Thus, to improve the desired functional properties, several starch modification methods have been developed [12]. One of the chemical modification methods that has been used to improve the functionality of native starch for DC is acid modification [13]. Thus, the present study is aimed at modifying the NEPS with hydrochloric acid and evaluating the modified starch as a potential directly compressible excipient in tablet formulations.

## 2. Materials and Methods

### 2.1. Materials

Fresh Ethiopian potato tubers were collected from Shashagalle kebele, Wolaita Zone, Southern Nations, Nationalities, and Peoples' Region, and authenticated by the National Herbarium of Ethiopia, Department of Plant Biology and Biodiversity Management, College of Natural Sciences and Computational Sciences, Addis Ababa University. Croscarmellose sodium (Rossmell Industries, India), disodium hydrogen orthophosphate dihydrate (TM MEDIA, Titan Biotech Ltd., India), magnesium stearate (Anhui Sunhere Pharmaceutical Excipients Co. Ltd., China), paracetamol powder (Anhui BBCA Likang Pharmaceutical Co. Ltd., China), and potassium dihydrogen orthophosphate (Loba Chemie Pvt. Ltd., Mumbai, India) were kindly donated by Addis Pharmaceutical Factory. Starch 1500® (Colorcon, France) was kindly donated by the School of Pharmacy, College of Health Sciences, Addis Ababa University. Hydrochloric acid (Loba Chemie Pvt. Ltd., India), sodium hydroxide (Neolab, Life Science Co., India), sodium metabisulphite (Sisco Research Laboratories Pvt. Ltd., India), and sodium chloride (Bulux Laboratories Ltd., India) were of analytical grade and used as received.

### 2.2. Methods

#### 2.2.1. Starch Extraction

Starch, from Ethiopian potato (EP) roots, was extracted following the method described by Assefa [8]. First, fresh EP roots were peeled, cut into smaller pieces, and blended using a home juice machine (Sinbo, SHB-3088, Philippines). The resulting paste was then suspended in distilled water containing 0.075% sodium metabisulphite (*w*/*v*). Then, the suspension was passed through a fine muslin cloth and the resulting filtrate was allowed to sediment. After the suspension had sediment, the supernatant was decanted and the sediment starch was repeatedly washed with 0.075% (*w*/*v*) sodium metabisulphite solution until the supernatant became clear (translucent). Then, it was washed several times with distilled water, dried in open air, milled, sieved using a 250 *μ*m mesh sieve, and stored in an airtight container for further use.

#### 2.2.2. Preliminary Study: Selection of Acid Modification Condition

To select the acid modification conditions, the effect of hydrolysis time and HCl concentration on the recovery yield of starch was studied. To establish the effect of hydrolysis time, two grams of NEPS (dry basis) was suspended in 10 mL of 6% (*w*/*v*) HCl solution at room temperature for 0, 2, 4, 6, 8, 10, and 14 days. Similarly, the effect of HCl concentration on the recovery yield of starch was studied by suspending two grams of NEPS (dry basis) in 10 mL of 0, 2, 4, 6, 8, 10, and 12% (*w*/*v*) HCl solution at room temperature for 8 days. The resulting suspension was then neutralized with 10% (*w*/*v*) of sodium hydroxide solution to terminate the hydrolysis. The starch slurry was then washed five times with distilled water until the pH of the filtrate became 7. The resulting starch was dried for 24 hours at 40°C in a hot air oven (Memmert, D-91129, Germany). This dried starch was then powdered using mortar and pestle, passed through a 250 *μ*m mesh sieve, and stored for further studies [14–19].

#### 2.2.3. Acid Modification of Ethiopian Potato Starch

The acid modification of NEPS was conducted by selecting the best result obtained in the preliminary study. Accordingly, 400 grams of NEPS (dry basis) was suspended in 600 mL of 6% HCl solution (*w*/*v*) at room temperature for 8 days. Then, the same procedure was followed as used in the preliminary study.

#### 2.2.4. Recovery Yield of the Acid Hydrolysis Process

The recovery yield (RY) was calculated using Equation (1) [19]:

(1) $$\begin{array}{c} \text{RY }\left(\%\right)=\frac{W_{\text{a}}}{W}\times 100, \end{array}$$

where *W* and *W*_a_ are the weight of the starch before and after acid hydrolysis, respectively.

#### 2.2.5. Spray Drying of Native and Acid-Modified Ethiopian Potato Starches

The spray drying process was carried out following the method described by Bilancetti et al. [20] using a spray dryer (Tall Form Spray Dryer, FT80, Armfield, USA). The inlet and outlet temperatures of the drying chamber were set at 180°C and 75°C, respectively. Finally, the spray-dried starch powders were passed through a 250 *μ*m mesh sieve and stored for further analysis.

### 2.3. Characterization of the Powder Properties

#### 2.3.1. X-Ray Diffraction Studies

The X-ray diffraction patterns of the NEPS and AMEPS samples were obtained using the method described by Nwokocha and Williams [21] in an X-ray diffractometer (DX-2700 SSC, China). The diffractometer was operated in the 2*θ*. The position of the peaks was analyzed using a copper target tube operating at 40 kV (30 mA) in the range of 3-64° of the 2*θ* with a microprocessor equipped with a single-crystal graphite monochromator.

#### 2.3.2. Density and Related Properties

For bulk and tapped density determination, a 100-gram sample of starch powder was carefully transferred into a 250 mL graduated glass measuring cylinder and the volume occupied was recorded. Then, it was placed onto a tap densitometer (Electrolab, ETD-1020, Navi Mumbai, India), which provides 300 taps/minute, and operated for one minute. Finally, the resulting volume was recorded, and the bulk density (*ρ*B) and tapped density (*ρ*T) were calculated from the bulk and tapped volumes using Equations (2) and (3), respectively. Carr's index (CI) and Hausner's ratio (HR) of the starch powders were calculated from the bulk and tapped densities using Equations (4) and (5), respectively [22].

(2) $$\begin{array}{c} \rho \text{B}=\frac{m}{V_{\text{B}}}, \end{array}$$

(3) $$\begin{array}{c} \rho \text{T}=\frac{m}{V_{\text{T}}}, \end{array}$$

where *m* is the mass of the sample and *V*_B_ and *V*_T_ are the volumes of the sample powder before and after tapping, respectively.

(4) $$\begin{array}{c} \text{CI}\left(\%\right)=\frac{\left(\rho \text{T}-\rho \text{B}\right)}{\rho \text{T}}\times 100, \end{array}$$

(5) $$\begin{array}{c} \text{HR}=\frac{\rho \text{T}}{\rho \text{B}}. \end{array}$$

#### 2.3.3. Flow Rate and Angle of Repose

Flow rate and angle of repose of the starch powders were determined by using a powder flowability tester (Pharma Test, PTG-S4, Apparatus AG, Germany). First, 50 g of powder, from each sample, was filled into the stainless steel funnel of the machine having a 15 mm outlet nozzle. The funnel was held at a height 10 cm above the base having a diameter of 10 cm. The stirring speed of the blade was held at 5 rpm. Then, the machine was allowed to operate, and the readings for the flow rate and angle of repose were recorded [22].

#### 2.3.4. Determination of Moisture Content

The moisture content of the powders was measured as per the method described by Olayemi et al. [23]. The percent moisture content was determined using

(6) $$\begin{array}{c} \text{Moisture content }\left(\%\right)=\frac{W_{\text{i}}-W_{\text{f}}}{W_{\text{i}}}\times 100, \end{array}$$

where *W*_i_ and *W*_f_ are the weights of the sample powder before and after drying, respectively.

#### 2.3.5. Determination of Swelling Power and Solubility

The swelling power (SP) and water solubility index (WSI) of the NEPS, AMEPS, and S1500® were determined following the methods described by Odeku and Picker-Freyer [24]. The starch sample (0.5 g) was first dispersed in 10 mL of distilled water in a dry preweighed centrifuge tube. The resulting starch suspensions were then placed on a thermostatically controlled water bath (HH-S4, 20225101, Germany) and kept at 25, 35, 45, 55, 65, 75, and 85°C for 30 minutes with frequent mixing at 5-minute intervals to keep the starch granules suspended. After the heating time is completed, the centrifuge tubes were removed from the water bath and left to cool to room temperature. Then, they were placed in a centrifuge machine (Table Top Centrifuge, PLC-03, Gemmy Industrial Corp., Taiwan) and centrifuged at 3000 rpm for 15 minutes. The supernatant was then carefully decanted into a dry preweighed Petri dish and dried in an oven at 120°C for 4 hrs. Finally, the WSI and swelling power (SP) were calculated from the weight of the dried supernatant (*W*_ds_) and the weight of the sediment remained after decantation (*W*_s_) using Equations (7) and (8), respectively.

(7) $$\begin{array}{c} \text{WSI}=2W_{\text{ds}}\times 100, \end{array}$$

(8) $$\begin{array}{c} \text{SP}=\frac{2W_{\text{s}}}{\left(100-\text{WSI}\right)}\times 100. \end{array}$$

#### 2.3.6. Determination of Moisture Sorption Patterns

The moisture sorption properties of the NEPS, AMEPS, and S1500® were investigated based on the method described by Gebre-Mariam and Schmidt (1996). Accordingly, the percentage of moisture sorbed (MS) by the samples was calculated from the weight of the samples before equilibration (*W*_b_) and after equilibration (*W*_a_) using

(9) $$\begin{array}{c} \text{MS}\left(\%\right)=\left(\frac{W_{\text{a}}-W_{\text{b}}}{W_{\text{b}}}\right)\times 100. \end{array}$$

#### 2.3.7. Drug-Excipient Compatibility Study

Drug-excipient compatibility was studied using a Fourier transform infrared spectroscopy (FT-IR) (IRPrestige-21, Shimadzu, Japan). This study was conducted for pure paracetamol and the mixture of paracetamol and acid-modified starch. These samples were then placed in the FT-IR machine and scanned in the wavenumber range from 500 to 4000 cm^−1^ at 25°C. The spectrum was obtained after the samples were scanned 20 times.

#### 2.3.8. Preparation of Tablet Formulations

All tablet formulations in this study were prepared using the DC method compressed at a constant compression force (15 ± 2 kN), using a single punch tablet machine (MINI Press II, Riva, Germany) fitted with a flat-faced punch and a die of 10 mm diameter. The prepared tablet formulations were evaluated and compared for compaction property, lubricant sensitivity, and dilution potential of the excipients used which are presented in the respective sections below.

#### 2.3.9. Evaluation of Compaction Property and Lubricant Sensitivity Study

The compaction property of NEPS, AMEPS, and S1500® was evaluated following the method described by Okunlola and Akingbala [25]. Accordingly, 300 mg compacts were prepared by compressing 99.5% of starch samples (298.5 mg) containing 0.5% magnesium stearate (1.5 mg) as a lubricant. These compacts were then evaluated for their crushing strength (CS), tensile strength (TS), percent friability (*f*(%)), and disintegration time (Dt). The lubricant sensitivity of the spray-dried NEPS, spray-dried AMEPS, and S1500® was evaluated as per the method described by Assen et al. [26]. Fifty grams of powder containing each excipient mixture with different concentrations of magnesium stearate, 0, 0.25% (125 mg), 0.5% (250 mg), 1% (500 mg), 1.5% (750 mg), and 2% (1000 mg), were blended for 5 minutes. Then, 300 mg blank tablets were prepared from each batch of the lubricated powder. Finally, these tablets were evaluated for their crushing strength, friability, and disintegration time.

#### 2.3.10. Dilution Potential

To evaluate the dilution potential of spray-dried NEPS and AMEPS, paracetamol was blended with each excipient at different ratios as indicated in Table 1. First, 40-gram batches of each formulation were prepared using the appropriate quantity of the excipients and blended for 10 minutes. Then, 0.5% magnesium stearate was added and further mixed for 5 minutes. Finally, 300 mg tablets were compressed from the lubricated blend [27, 28]. The prepared paracetamol tablets were evaluated for their tablet properties.

### 2.4. Evaluation of the Prepared Tablets

#### 2.4.1. Hardness Test and Tablet Dimensions

The crushing strength (CS) of tablets was determined using a hardness tester (Pharma Test, PTB 311E, D-63512, Germany). Ten tablets were randomly selected from each formulation, and the CS of each tablet was measured [29]. The tablet thickness and diameter were simultaneously measured while the hardness test was conducted using a hardness tester. Then, the radial tensile strength (TS) was calculated using Equation (10) [29].

(10) $$\begin{array}{c} \text{TS}=\frac{2\text{CS}}{\left(\pi DT\right)}, \end{array}$$

where *D* is the diameter and *T* is the thickness.

#### 2.4.2. Friability

Ten tablets from each batch were weighed and placed in a plastic chamber of a friability tester (Pharma Test, PTF 10E, D-63512, Hamburg, Germany) and operated at a revolution speed of 25 rpm for 4 min. The tablets were then collected, dedusted, and weighed, and the percentage weight loss was calculated and displayed as percentage friability [22].

#### 2.4.3. Disintegration Time

The disintegration test was carried out using a USP disintegration tester (Pharma Test, PTZ S, D-63512, Germany) for each batch taking six tablets randomly. Each tablet was placed in a disintegration apparatus containing 900 mL of distilled water and maintained at 37 ± 2°C. The machine was then allowed to operate, and the time taken for complete disintegration and to pass through the mesh was recorded as the disintegration time.

#### 2.4.4. Dissolution Study

The dissolution tests were performed according to the USP/NF [22] specifications for immediate-release dosage forms using type II dissolution apparatus (paddle) (Pharma Test, PTW S 820 D, D-63512, Hamburg, Germany) at a paddle rotation speed of 50 rpm. First, six tablets from each formulation were placed in the dissolution vessel filled with 900 mL phosphate buffer (pH 5.8) solution which was maintained at 37 ± 0.5°C. Then, at predetermined intervals, at 5, 10, 15, 20, 30, 45, and 60 minutes, samples were withdrawn, filtered, and appropriately diluted, and absorbance readings were taken at *λ*_max_ of 243 nm. Each withdrawn sample was replaced with an equal volume of fresh dissolution medium which kept at the same temperature.

### 2.5. Data Analysis

Statistical analysis was performed using analysis of variance (ANOVA) with SPSS statistical software package (IBM SPSS Statistics 20). Origin8® software (Origin Pro 8 Corporation, USA) was used to plot graphs. At a 95% confidence interval, *p* values of ≤0.05 were considered statistically significant. All the data measured and reported are averages of a minimum of triplicate measurements, and the values are expressed as mean ± standard deviation.

## 3. Results and Discussion

### 3.1. Selection of Acid Modification Condition

**Figure 2:**
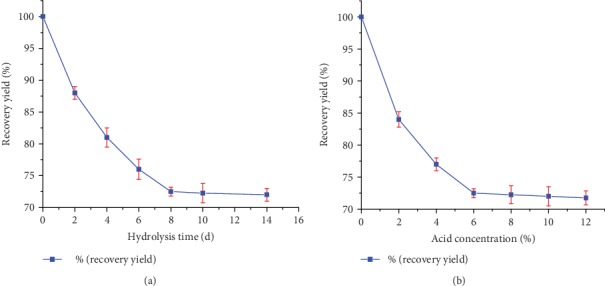
(a) Recovery yield of Ethiopian potato starch as a function of acid hydrolysis time at HCl concentration of 6% (*w*/*v*) and temperature of 25°C. (b) Recovery yield of Ethiopian potato starch as a function of acid concentration at hydrolysis time of 8 days and room temperature of 25°C.

The acid recovery yield of EP starch at different hydrolysis times is presented in Figure 2(a). From this figure, it can be observed that in the first 8 days, there was a fast hydrolysis while slow hydrolysis was observed after 8 days. The acid recovery yield of AMEPS was 72.5% after 8 days of acid hydrolysis and remained almost constant at 14 days (72%).  Figure 2(b) depicts the acid recovery yield as a function of acid concentration. Accordingly, the recovery yield decreased significantly as the HCl concentration increased to 6%. However, further increment of the concentration to 12% did not result in to appreciable changes, 72.5% at 6% versus 71.75% at 12% of HCl concentration. Therefore, the modification of starch was conducted using 6% of HCl for hydrolysis time of 8 days.

### 3.2. Powder Characterizations

#### 3.2.1. Crystallinity of the Starches

**Figure 3:**
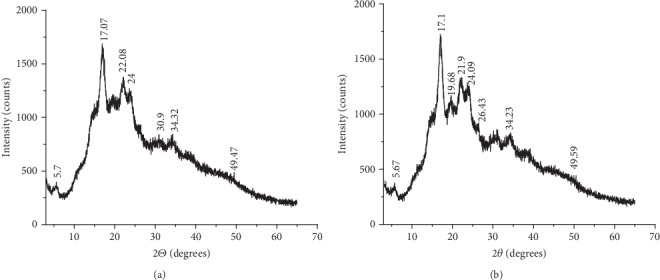
X-ray powder diffraction patterns of NEPS (a) and AMEPS (b).

The X-ray diffractograms of NEPS and AMEPS are presented in Figures 3(a) and 3(b), respectively. Accordingly, the NEPS exhibited X-ray diffraction peaks at around 17°, 22°, 22.5°, and 24° 2*θ* with other minor peaks (5.794°, 30.915°, 34.326°, and 49.457°). The presence of strong diffraction peaks at around 17° and small peaks at around 5.6°, 20°, 22°, and 24° 2*θ* angles indicated that the NEPSs have B-type crystals which are in line with other tuber starches [19]. Similar peaks were also observed in the AMEPS which suggest that the acid modification did not bring about any transition of the crystal type [30, 31]. However, comparing the peaks, the AMEPS showed slightly sharper and intense peaks at 17.156°, 19.584°, 21.899°, and 22.535° at 2*θ*. This may indicate that the internal structure of the starch granules had changed and resulted in a more crystalline structure [18, 19, 32].

#### 3.2.2. Density and Related Properties

The density and related properties of the NEPS, AMEPS, and S1500® are presented in Table 2. The bulk density of spray-dried AMEPS was comparable with that of S1500® while the AMEPSs have significantly higher values compared to the NEPSs (*p* < 0.05). This higher bulk density is advantageous in tableting due to a reduction in the fill volume of the die [33]. The changes in particle size and shape of the starches during the acid modification and spray drying process might be responsible for the differences in the density value [34].

The air- and spray-dried NEPS exhibited significantly higher CI and HR values compared to the AMEPSs (the oven- and spray-dried) (*p* < 0.05) indicating poor flow property of the NEPS. The CI and HR of spray-dried AMEPS were found to be 11.48% and 1.13, respectively, indicating good powder flow properties. This result indicated that spray drying had significantly improved its flow properties (*p* < 0.05). This might be due to the presence of greater amounts of spherical-shaped particles produced during the spray drying process. Similarly, spray drying decreased the CI and HR of both the NEPS and AMEPS. On the other hand, S1500® showed excellent flow properties which might be due to its higher densities and particle size [5].

#### 3.2.3. Angle of Repose and Flow Rate

The results of the angle of repose for the spray-dried NEPS indicated poor flow characteristics while the spray-dried AMEPS showed excellent powder flowability. The air-dried NEPS and oven-dried AMEPS, on the other hand, could not flow through an opening. This indicated that the spray drying had improved the flow properties of both the NEPS and AMEPS. The flow improvement, however, was significantly higher for the AMEPS compared to the NEPS (excellent versus poor flowability). The difference could be attributed to the acid modification of the NEPS prior to the spray drying. On the other hand, no significant difference was observed in the angle of repose of spray-dried AMEPS and S1500® indicating excellent powder flowability. The flow rate, as can be noticed in Table 2, was in line with the results of the angle of repose. It was ranked in ascending order as spray-dried NEPS<spray-dried AMEPS<S1500® (*p* < 0.05). The flow rate of the air-dried NEPS and oven-dried AMEPS could not be determined for the same reason mentioned above in the angle of repose.

#### 3.2.4. Moisture Content

The moisture contents of the NEPSs, AMEPSs, and S1500® are presented in Table 3. Accordingly, the spray-dried AMEPS had a significantly minimum moisture content compared to the spray-dried NEPS (*p* < 0.05). This could be attributed to the difference in the relative crystallinity of the starches. However, no significant difference was observed in moisture content within the NEPSs and similarly between the AMEPSs (*p* > 0.05). This result indicated that the spray drying process did not bring about any significant change in the moisture content of the NEPS and AMEPS (*p* > 0.05) in contrast to the study done by Assen et al. [26] and Tessema et al. [34]. On the other hand, the moisture content of S1500® was significantly lower than that of the other counterparts. This could be related to the production process of partially pregelatinized corn starch under different pretreatment conditions [35]. Generally, the maximum moisture content recommended for safe storage of starch is below 13% (*w*/*w*) [36].

#### 3.2.5. Swelling Power and Solubility

**Figure 4:**
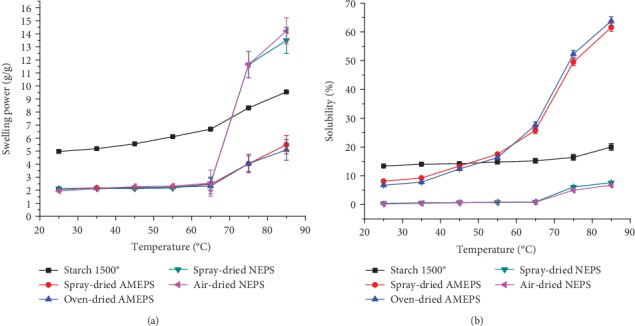
Swelling power (a) and solubility (b) of NEPS, AMEPS, and S1500® at different temperatures.

The SP of all the starch powders studied is depicted in Figure 4(a). As can be seen from this figure, the SP of the NEPSs and AMEPSs were comparable up to 65°C while S1500® was significantly higher (*p* < 0.05). The lower SP of the NEPS and AMEPS may be related to the strong bonding forces within the granules making the starches relatively resistant to swell [37]. In contrast, S1500® had highly disorganized starch granules due to the pregelatinization process [5, 38]. On the other hand, above 65°C, the SP for all starches studied showed a significant increase which might be due to the macromolecular disorganization and crystalline structure disruption [24]. It could also be noticed that, beyond this temperature, the SP of the NEPS is significantly higher than the AMEPS (*p* < 0.05). This could be attributed to the reduction in chain length and hydrolysis of the amorphous parts of the starch granules as a result of the acid modification. Amylopectin chain length is one of the factors that affects SP [39].

The solubility of the NEPSs, AMEPSs, and S1500® increased with increasing temperature as can be noticed in Figure 4(b). However, only the AMEPSs exhibited a significant increase (*p* < 0.05) which might be due to the changes in the structure of starch granules and amylose leaching because of acid hydrolysis [40]. Due to this, the AMEPSs showed higher solubility at all temperature points compared to the NEPS. On the other hand, AMEPS showed lower solubility than S1500® up to 55°C. Generally, the acid modification favoured disintegration and solubilisation rather than swelling of the starch. Furthermore, no significant change was observed in the SP and solubility of NEPS and AMEPS before and after spray drying (*p* > 0.05). Thus, the drying methods had no significant effect in this case.

#### 3.2.6. Moisture Sorption Property

**Figure 5:**
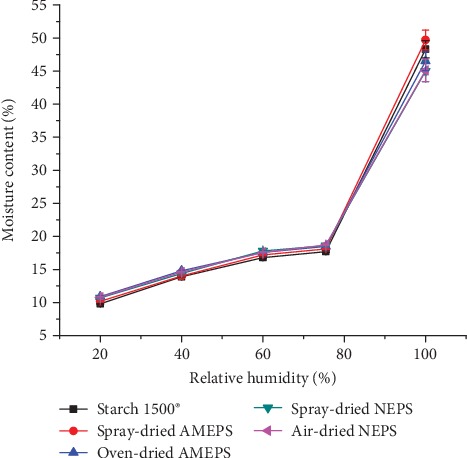
Moisture sorption patterns of NEPS, AMEPS, and S1500® at different RH.

The moisture sorption profiles of the NEPSs, AMEPSs, and S1500® are depicted in Figure 5. Accordingly, their moisture sorption profiles were comparable without a significant difference at all RH value (*p* > 0.05). All the starches studied showed a slight increase in their moisture sorption between 20 and 60% RH. However, beyond 75.5% RH, the moisture sorption significantly increased. This sharp increase above 75.5% RH could be attributed to change in mechanism of sorption. This reinforces the necessity for moisture avoidance during storage [41].

#### 3.2.7. Drug-Excipient Compatibility Study

**Figure 6:**
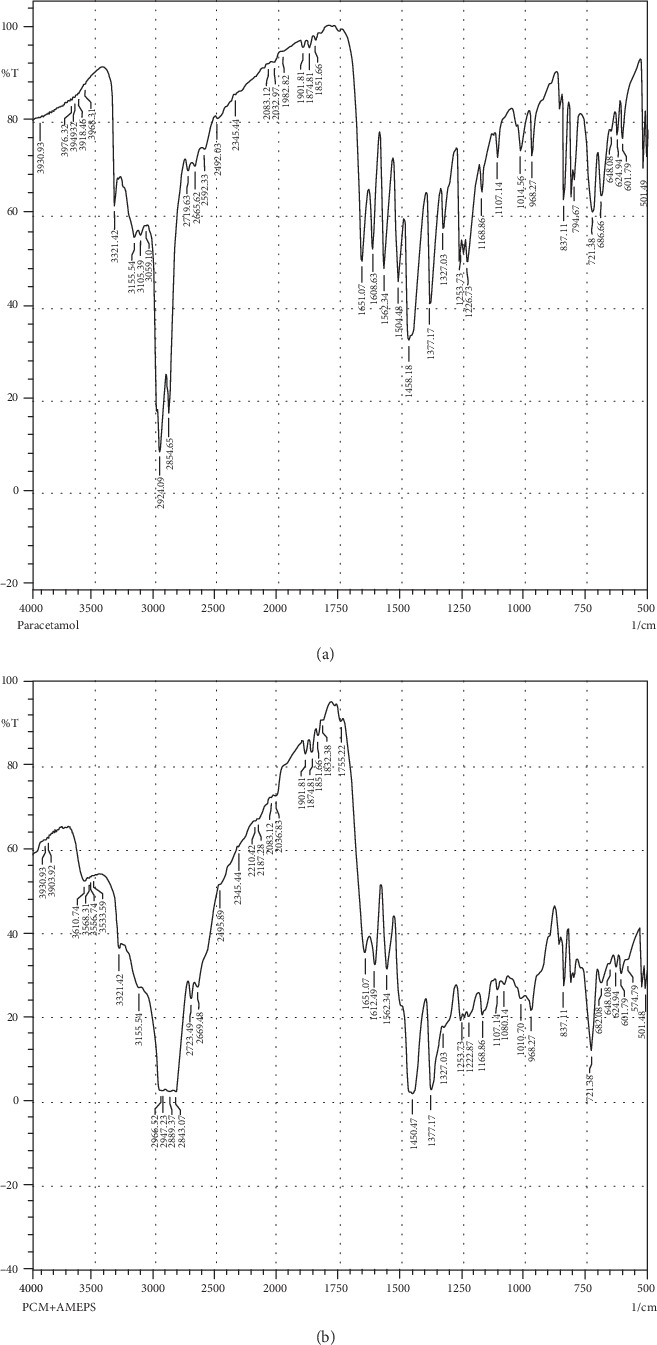
FT-IR spectra of paracetamol (a) and the physical mixture of paracetamol and AMEPS (b).

The FT-IR spectrum results of paracetamol and its physical mixture with AMEPS are depicted in Figures 6(a) and 6(b), respectively. Accordingly, the observed characteristic peaks of pure paracetamol are at the 3321.42 cm^−1^ (O-H stretching), 3155.54 cm^−1^ (N-H stretching), and 1651.07 cm^−1^ (C=O (amide) stretching) functional groups, whereas amide II band stretching at 1562.34 cm^−1^, OH deformation -C-O stretching at 1253.73 cm^−1^, C-H stretching at 3000 cm^−1^ to 2800 cm^−1^, and aromatic ring stretching vibration band at 1608.63 cm^−1^ were also observed ([42, 43]; Bashar, 2010). The presence of these characteristic peaks of paracetamol in the physical mixture of paracetamol and AMEPS and the absence of any major shift indicated that there is no interaction between them.

#### 3.2.8. Powder Compaction Property

**Figure 7:**
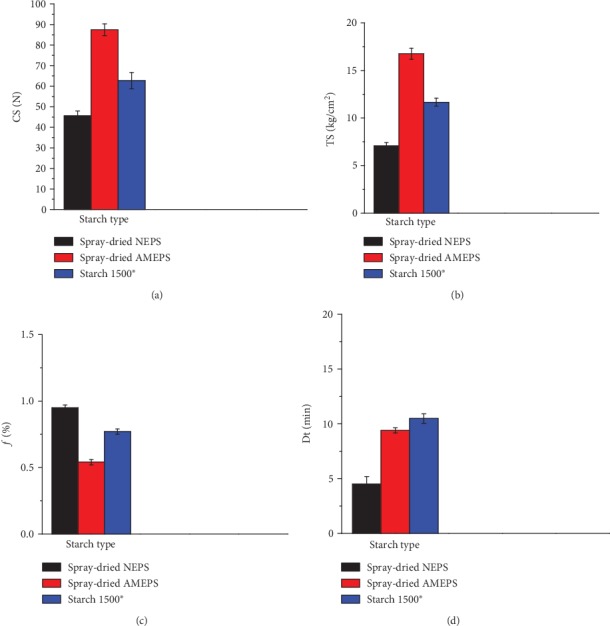
Crushing strength (a), tensile strength (b), friability (c), and disintegration time (d) of blank tablets prepared from spray-dried NEPS, spray-dried AMEPS, and S1500®.

The tablet characteristics of the blank tablets prepared for the compaction study are presented in Figures 7(a)–7(d). The CS and TS of these tablets were ranked in the order of spray-dried AMEPS>S1500®>spray-dried NEPS (*p* < 0.05). The higher CS and TS of the AMEPS tablets indicated that it has a higher compactability which could be attributed to the acid modification. Acid hydrolysis cleaves the amorphous region and increases the relative crystallinity. These crystalline regions could be forced closer with increased intermolecular force during compression resulting in a higher tablet hardness [14]. The results in the AMEPS compacts are in agreement with other similar studies reported [40, 44, 45].

The resistance to friability and disintegration time of the blank tablets prepared from NEPS, AMEPS, or S1500® are in line with the values of their hardness. This is to mean that their values increased with increasing tablet hardness. Hence, the friability of the tablets in this study was ranked in descending order as spray-dried NEPS>S1500®>spray-dried AMEPS (*p* < 0.05). On the other hand, the disintegration time was ranked in the order of S1500®>spray-dried AMEPS>spray-dried NEPS (*p* < 0.05). The spray-dried AMEPS tablets showed prolonged disintegration time than the spray-dried NEPS which could be attributed to their higher hardness. Generally, all the blank tablets were able to disintegrate into their primary particles within 15 minutes.

### 3.3. Lubricant Sensitivity Study

#### 3.3.1. Effect of Lubricant Concentration on Crushing Strength and Friability

**Figure 8:**
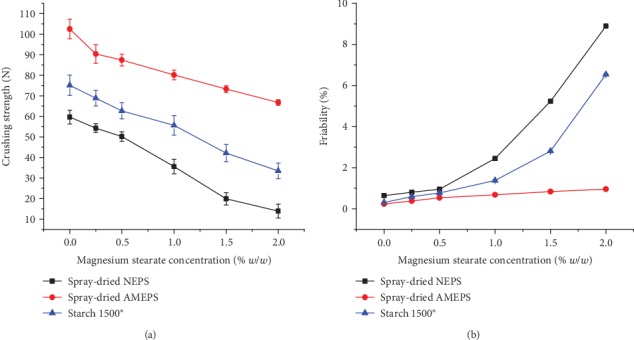
Crushing strength (a) and friability (b) of spray-dried NEPS, spray-dried AMEPS, and S1500® tablets at different concentrations of magnesium stearate.

The CS of all the prepared tablets for lubricant sensitivity studies generally decreased as the concentration of magnesium stearate increased (Figure 8(a)). This could be due to the nature of lubricant films inhibiting the interparticle bond [46]. Despite this, tablets of the spray-dried AMEPS had significantly higher CS than those of the spray-dried NEPS and S1500® at all magnesium stearate concentrations (*p* < 0.05) with an average CS of 66.73, 13.9, and 33.46 N, respectively, at 2% lubricant concentration. On the other hand, the spray-dried NEPS and S1500® produced soft tablets with an average CS of 13.9 and 33.46 N, respectively, at 2% magnesium stearate concentration. On the other hand, as depicted in Figure 8(b), the friability of all the tablets prepared increased with increasing magnesium stearate concentration which is in line with the decline in CS. Although a general trend of increasing friability was observed, the tablets of spray-dried AMEPS were within the acceptable friability range (<1%) at all levels of magnesium stearate concentration. On the other hand, the spray-dried NEPS and S1500® tablets showed an acceptable friability value only up to 0.5% magnesium stearate concentration.

#### 3.3.2. Effect of Lubricant Concentration on Disintegration Time

**Figure 9:**
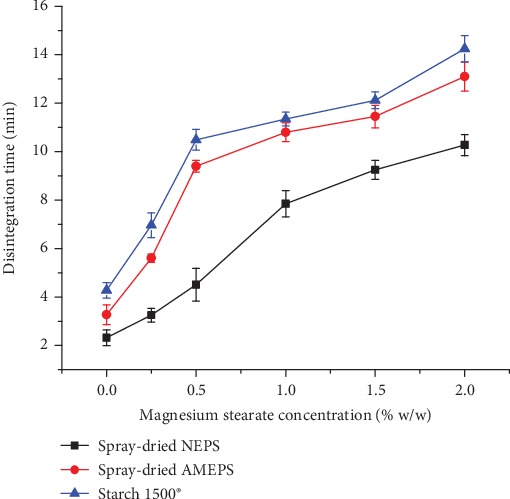
Disintegration time of spray-dried NEPS, spray-dried AMEPS, and S1500® tablets at different concentrations of magnesium stearate.

Figure 9 depicts the effect of lubricant concentration on the disintegration time of tablets. Accordingly, the tablet disintegration time increased with the concentration of magnesium stearate. This is because hydrophobic lubricants such as magnesium stearate form a hydrophobic film around particles which hinders water penetration into tablets [47]. The disintegration time for tablets of the spray-dried AMEPS and S1500® was comparable with no significant difference (*p* > 0.05) at all levels of magnesium sterate concentration. However, the former possesses a shorter disintegration time even without considering its higher CS. The longest disintegration period observed in the tablets made of S1500® could be related to the formation of a gel-like layer, which is formed in combination with water preventing their disintegration. The shortest disintegration time was registered for tablets of spray-dried NEPS at all levels of magnesium stearate which could be attributed to their lower CS.

### 3.4. Dilution Potential

#### 3.4.1. Tablet Hardness

The hardness of tablets could be expressed in terms of CS and TS which are related to the mechanical strength of tablets [29]. Crushing strength shows the ability of tablets to withstand stress and resist breakage [4]. Unlike CS, employment of TS allows the dimensions of the tablets to be taken into account [48]. Thus, TS was devised for the comparison of the mechanical strength of the prepared paracetamol tablets.

**Figure 10:**
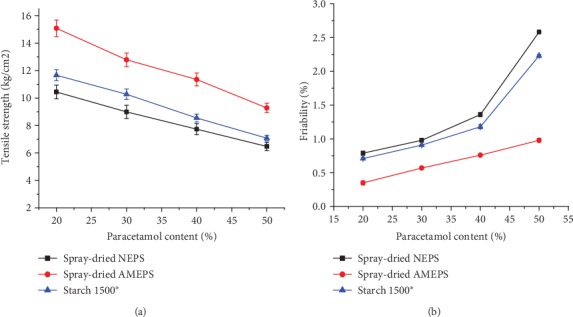
The tensile strength (a) and friability (b) of tablets formulated from spray-dried NEPS, spray-dried AMEPS, and S1500® at different paracetamol content.

The TS of the paracetamol tablets prepared from NEPS, AMEPS, and S1500® were given in Figure 10(a). Tensile strength decreased with increasing paracetamol concentration in all formulations (*p* < 0.05). This could be related to the poor compressibility characteristic and high elastic recovery of paracetamol. The tensile strength of tablets from the AMEPS was significantly higher than both the NEPS and S1500® at all levels of paracetamol concentration (*p* < 0.05). This implies that the AMEPS underwent a higher plastic deformation that predominates the disruptive elastic recovery of paracetamol [27]. The results for the tensile strength are in line with the values of the crushing strength. The values of crushing strength at 50% of paracetamol content were 37.6, 40.2, and 51.5 N for the spray-dried NEPS, S1500®, and spray-dried AMEPS, respectively. Thus, only the spray-dried AMEPS could hold up to 50% of paracetamol with acceptable crushing strength, greater than 50 N [28]. The NEPS and S1500® produced acceptable tablet strength only up to 30% of paracetamol content.

#### 3.4.2. Friability

The friability test results of all paracetamol tablet formulations are depicted in Figure 10(b). Accordingly, the friability results were ranked in increasing order of spray-dried AMEPS<S1500®<spray-dried NEPS with a significant difference (*p* < 0.05). Tablets of the spray-dried AMEPS fulfilled the acceptance criteria for friability at all paracetamol contents while tablets of the spray-dried NEPS and S1500® fulfilled this specification only up to 30%. The higher friability values of spray-dried NEPS and S1500® at higher paracetamol concentration could be due to their low tablet mechanical strength as discussed above. There is an inverse relationship between tablet mechanical strength and friability in such a way that the latter declines as the former increases [49].

#### 3.4.3. Disintegration Time

**Figure 11:**
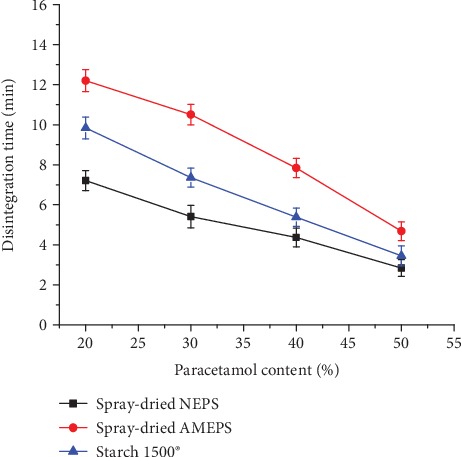
The disintegration time of tablets formulated from spray-dried NEPS, spray-dried AMEPS, and S1500® at different paracetamol loading.

The disintegration time of all tablets formulated from the spray-dried NEPS, spray-dried AMEPS, and S1500® decreased significantly with increased content of paracetamol (*p* < 0.05) (Figure 11). This could be attributed to a poorly compactable nature of paracetamol which makes the prepared tablet weak and easy for penetration of water into the bulk. Generally, the disintegration time was in the order of spray-dried AMEPS>S1500®>spray-dried NEPS at all paracetamol content. The longer disintegration time observed for tablets of the spray-dried AMEPS at all paracetamol concentrations could be related to their superior mechanical strength. In addition, AMEPS showed a lower SP which probably could be another factor for the prolongation of the disintegration time. In spite of all these differences, all the paracetamol tablet formulations fulfilled the pharmacopoeial specification for the disintegration time of conventional tablets (<15 minutes) [22].

#### 3.4.4. Dissolution Study of Paracetamol Tablets

**Figure 12:**
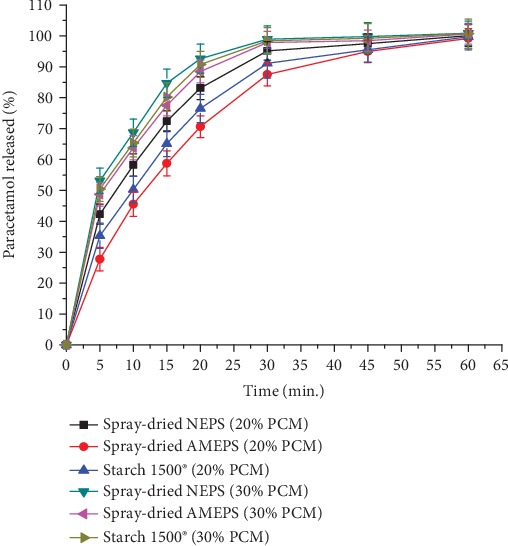
The dissolution profile of tablets prepared from the spray-dried NEPS, spray-dried AMEPS, and S1500® at 20% and 30% paracetamol content.

The dissolution profiles of all the selected paracetamol tablets of the spray-dried NEPSs, spray-dried AMEPSs, and S1500®s are presented in Figure 12. Accordingly, the percentage of drug released at 30 minutes from the tablets containing 20% of paracetamol was in the order of NEPS (95.14 ± 3)>S1500® (91.12 ± 4.8)>spray-dried AMEPS (87.54 ± 3.7) without a significant difference (*p* > 0.05). This is in line with the disintegration time of the respective tablets. A similar trend was also observed for tablets containing 30% of paracetamol. In both concentrations of paracetamol, the prepared tablet formulations fulfilled the dissolution specification set by the USP 30/NF 25 [22], which is releasing more than 80% of their content within 30 minutes.

### 3.5. Limitation of the Study

The main limitation of this study was only a single compression force was used in the powder compaction property study. The compaction property would have been better explained if compression force of 3-4 levels versus hardness profile was measured.

## 4. Conclusions

The result of this study indicates that acid modification improves the flow properties of the native Ethiopian potato starch. This improvement was further enhanced by the spray drying process. In addition, the spray-dried AMEPS showed a higher compactability compared to both the spray-dried NEPS and S1500® by yielding tablets of higher mechanical strength and acceptable friability values. The spray-dried AMEPS was also able to incorporate up to 2% of magnesium stearate satisfying the tablet specifications while the spray-dried NEPS and S1500® incorporated only up to 0.5%. Similarly, it was noticed that only the spray-dried AMEPS was capable of incorporating up to 50% of paracetamol content with acceptable pharmacopoeial specifications. From the perspective of the above mentioned results, spray-dried AMEPS could be a potential alternative candidate as a directly compressible tablet excipient.

## Figures and Tables

**Table 1:** Paracetamol tablet formulations containing spray-dried NEPS, spray-dried AMEPS, and S1500® as a directly compressible excipient at four levels of concentration.

Ingredients (%)	Formulations											
	F1	F2	F3	F4	F5	F6	F7	F8	F9	F10	F11	F12
Paracetamol	20	30	40	50	20	30	40	50	20	30	40	50
S_d_NEPS	75.5	65.5	55.5	45.5	—	—	—	—	—	—	—	—
S_d_AMEPS	—	—	—	—	75.5	65.5	55.5	45.5	—	—	—	—
S1500®	—	—	—	—	—	—	—	—	75.5	65.5	55.5	45.5
CCS	4	4	4	4	4	4	4	4	4	4	4	4
Magnesium stearate	0.5	0.5	0.5	0.5	0.5	0.5	0.5	0.5	0.5	0.5	0.5	0.5

CCS: croscarmellose sodium. S_d_NEPS and S_d_AMEPS stand for spray-dried native and acid-modified Ethiopian potato starch, respectively.

**Table 2:** Densities and powder flow properties of NEPS, AMEPS, and S1500®.

Powder properties	Air-dried NEPS	Spray-dried NEPS	Oven-dried AMEPS	Spray-dried AMEPS	Starch 1500®
Bulk density (g/mL)	0.40 ± 0.02^a^	0.48 ± 0.02^b^	0.50 ± 0.01	0.62 ± 0.02	0.64 ± 0.01
Tapped density (g/m)	0.55 ± 0.01	0.63 ± 0.01	0.57 ± 0.02	0.70 ± 0.01	0.71 ± 0.02
Hausner's ratio	1.37 ± 0.02^a^	1.31 ± 0.02^b^	1.15 ± 0.01	1.13 ± 0.01	1.10 ± 0.02
Carr's index (%)	27.43 ± 0.29^a^	23.93 ± 0.23^b^	12.93 ± 1.92	11.48 ± 0.10	9.90 ± 0.08
Angle of repose (^o^)	^∗^	45.40 ± 1.91	^∗^	27.07 ± 0.25	26.50 ± 1.73
Flow rate (g/sec)	^∗^	0.62 ± 0.38	^∗^	5.31 ± 0.06	10.56 ± 0.25

^∗^Angle of repose and flow rate could not be determined. ^a^*p* < 0.05 vs. oven-dried AMEPS. ^b^*p* < 0.05 vs. spray-dried AMEPS.

**Table 3:** The moisture contents of NEPS, AMEPS, and S1500®.

Starches	Moisture content (%)
Air-dried NEPS	15.35 ± 0.050
Spray-dried NEPS	15.30 ± 0.050
Oven-dried AMEPS	11.42 ± 0.076
Spray-dried AMEPS	11.82 ± 0.005^a^
S1500®	10.82 ± 0.005

^a^ *p* < 0.05 vs. spray-dried NEPS.

## Data Availability

The data used to support the findings of this study are included within the article.
